# A cell death assay in barley and wheat protoplasts for identification and validation of matching pathogen AVR effector and plant NLR immune receptors

**DOI:** 10.1186/s13007-019-0502-0

**Published:** 2019-10-24

**Authors:** Isabel M. L. Saur, Saskia Bauer, Xunli Lu, Paul Schulze-Lefert

**Affiliations:** 1 Department of Plant-Microbe Interactions, Max Planck Institute for Plant Breeding Research, 50829 Cologne, Germany; 2 Cluster of Excellence on Plant Sciences, 40225 Düsseldorf, Germany; 3 Present Address: Department of Plant Pathology, College of Plant Protection, China Agricultural University, Beijing, 100193 China

**Keywords:** NLR-type immune receptor, Pathogen avirulence effector, Cell death, Race-specific disease resistance, Barley, Wheat, Leaf protoplasts

## Abstract

**Background:**

Plant disease resistance to host-adapted pathogens is often mediated by host nucleotide-binding and leucine-rich repeat (NLR) receptors that detect matching pathogen avirulence effectors (AVR) inside plant cells. AVR-triggered NLR activation is typically associated with a rapid host cell death at sites of attempted infection and this response constitutes a widely used surrogate for NLR activation. However, it is challenging to assess this cell death in cereal hosts.

**Results:**

Here we quantify cell death upon NLR-mediated recognition of fungal pathogen AVRs in mesophyll leaf protoplasts of barley and wheat. We provide measurements for the recognition of the fungal AVRs *AvrSr50* and *AVR*_*a1*_ by their respective cereal NLRs *Sr50* and *Mla1* upon overexpression of the *AVR* and *NLR* pairs in mesophyll protoplast of both, wheat and barley.

**Conclusions:**

Our data demonstrate that the here described approach can be effectively used to detect and quantify death of wheat and barley cells induced by overexpression of *NLR* and *AVR* effectors or *AVR* effector candidate genes from diverse fungal pathogens within 24 h.

## Background

Monocotyledonous wheat (*Triticum durum*, *Triticum aestivum*) and barley (*Hordeum vulgare*) are important crops worldwide and diseases caused by infectious pathogens threaten their cultivation. The genomes of bacterial, fungal, and oomycete plant pathogens encode numerous virulence factors (so-called effectors) that either interfere with the plant immune system or manipulate the metabolism of their hosts, ultimately leading to disease development and proliferation of the pathogen [1]. Disease resistance to host-adapted pathogens is often mediated through the recognition of pathogen effectors by plant-encoded nucleotide-binding and leucine-rich repeat receptors (NLR) [2]. NLRs detect either the effector structure or effector-mediated modifications of additional host proteins (guards or decoys) [3, 4]. Effectors recognized by NLRs are termed avirulence (AVR) effectors. Usually, NLR-mediated AVR effector recognition is associated with a rapid host cell death at the site of attempted infection, called the hypersensitive response.

Molecular isolation of *NLRs* and introgression of the corresponding genes into economically relevant crop varieties can contribute significantly to minimizing losses due to crop disease in modern agriculture. Similarly, isolation of pathogen effectors can afford insights into their roles in disease development in susceptible hosts. Successful identification of *AVRs* and *NLRs* depends on molecular and genetic verification of AVR recognition by host plant NLRs, but this is challenging to evaluate in cereal hosts.

The development of the method described here was motivated by the need for a method to test pathogen *AVR* candidates by rapidly assaying cell death mediated by matching NLR/AVR pairs in barley and wheat hosts, whilst avoiding the limitations of existing protocols. An existing method most closely resembling the natural delivery of effectors into plant host cells during pathogen infection is the delivery of pathogen effectors into resistant hosts via the bacterial type-III secretion system [5]. Although successful in one case [6, 7], type III secretion of fungal AVRs into cereals is not used extensively and failed to identify *Bgh AVR*_*a1*_ and *AVR*_*a13*_ [8] for unknown reasons.

The most commonly used alternative to bacterial type III-mediated AVR delivery into host cells is *in planta* co-expression of *AVR* and matching *NLR* genes. Generation of transgenic plants expressing pathogen effectors and subsequent crossing to plants encoding matching NLR resistance specificities can be performed to determine AVR-dependent NLR activation [8, 9]. Cell death in successful crosses is usually determined by seedling lethality and/or plant growth retardation. Yet, the method ideally requires the availability of AVR-specific antibodies or epitope-tag fusions of pathogen effectors for immunoblot detection, as *AVR* gene expression and steady-state levels of the encoded protein can substantially vary between individual transgenic lines [8]. However, epitope fusion may compromise the avirulence activity of effectors. Considering the large expenditure of time needed (several months) and the difficulty in generating stable transgenic cereal plants, the use of transient expression systems is to be preferred.

Virus-mediated overexpression (VOX) could serve as transient gene expression system to screen *AVR* candidates in resistant lines when the host *NLR* has not been molecularly isolated. In comparison to previously described viral expression vectors [10, 11], the recently described Foxtail mosaic virus (FoMV)-based expression system has been shown to establish systemic infection with reduced chlorotic/necrotic mosaic symptoms in infected monocotyledonous leaves. The size of genes expressed via VOX is limited, but FoMV appears to be suitable for the expression of *AVR* genes as fluorescent GFP protein was expressed comprising 238 amino acids (aa) in wheat and GUS protein consisting of 600 aa in maize [12]. Nevertheless, the FoMV system is limited to plant accessions susceptible to FoMV [12].

Transient *Agrobacterium*-mediated heterologous overexpression of *NLR/AVR* pairs in *Nicotiana benthamiana* or *Nicotiana tabacum* is widely used and allows direct visualization of cell death on the leaves a few days after transient transformation with *NLR* and *AVR* constructs. Although it is a convenient tool in terms of time needed and ease of handling, the method has numerous limitations: Firstly, overexpression of some *NLRs* alone can already elicit *AVR*-independent cell death responses in a heterologous system due to high *NLR* expression levels or the lack of cell death regulating components [13–15]. Secondly, the heterologous nature of the system can limit expression, protein levels and the activity of both NLR and AVR, thereby again requiring epitope fusions of both NLR and AVR to determine protein stability; this, in turn, may compromise AVR/NLR function [16]. For each *NLR/AVR* pair, transformation levels and ratios, as well as epitope fusions may require extensive optimisation in the *N. benthamiana* system [17, 18]. For example, disproportionate experimental efforts were needed to detect specific cell death mediated by the MLA1/AVR_A1_ pair in *N. benthamiana* and we found that the detection of this read-out necessitated C-terminal fusion of AVR_A1_ to the monomeric yellow fluorescent protein [17] in this heterologous system [8, 17]. Additionally, many NLRs rely on host lineage-specific proteins for AVR recognition (indirect recognition) and these proteins may be absent or too diverged in *Nicotiana ssp.* Thus, a lack of cell death in the heterologous *Nicotiana* systems might not necessarily be because of a lack of AVR-mediated NLR activation but may instead be due to the heterologous nature of the system. One example is the lack of cell death upon co-expression of the matching *Bgh AVR*_*a9*_—barley *Mla9* pair in heterologous *N. benthamiana*, while cell death is induced in homologous barley [17].

As such, there was a need for a homologous transient expression system to measure AVR-specific cell death mediated by cereal NLRs. We had aimed to establish such an assay for wheat and barley and found the transfection of mesophyll protoplasts as suitable. We attempted to use mesophyll protoplasts derived from barley and wheat leaves for rapidly assaying cell death mediated by matching cereal NLR/fungal AVR pairs. For this, we first significantly modified various steps in existing cell transfection procedures [19] to allow the successful transfection of multiple binary plasmids into wheat and barley mesophyll protoplasts. We use epidermal peeling for the exposure of mesophyll leaf cells, optimized the age of plant and tissue for protoplast isolation and the size, amount and ratio of plasmids transfected as well as buffer compositions (methods). The scheme can be used to screen for the identification and verification of pathogen effector candidates [8, 17] but has not yet been applied to wheat.

We show that our method also proved successful for wheat, at least when overexpressing *NLR/AVR* pairs, as we could quantify cell death upon recognition of the stem rust fungus *Puccinia graminis* f. sp. *tritici* (*Pgt*) effector *AvrSr50* [20] by its matching NLR *Sr50* [21], both in wheat and in barley mesophyll protoplasts. We depict how mesophyll protoplasts derived from barley and wheat leaves, and possibly leaves from other cereals, can be transfected and screened for the identification and verification of pathogen effector candidates derived from two unrelated fungal pathogens. Our results also demonstrate that the here described method allows assessment of NLR activity following *NLR* transfer in another cereal plant species. The approach is thus suitable for the assessment of NLR function in diverse host cultivars or other cereal plant species. This is of particular interest when stacking/pyramiding NLRs in single plants. NLR stacking/pyramiding should provide for durable disease resistance that cannot be easily overcome by pathogens, yet AVR-mediated cell death of some NLRs is impaired by the co-occurrence of other NLRs for largely unknown reasons [22–24].

## Results

**Fig. 1:**
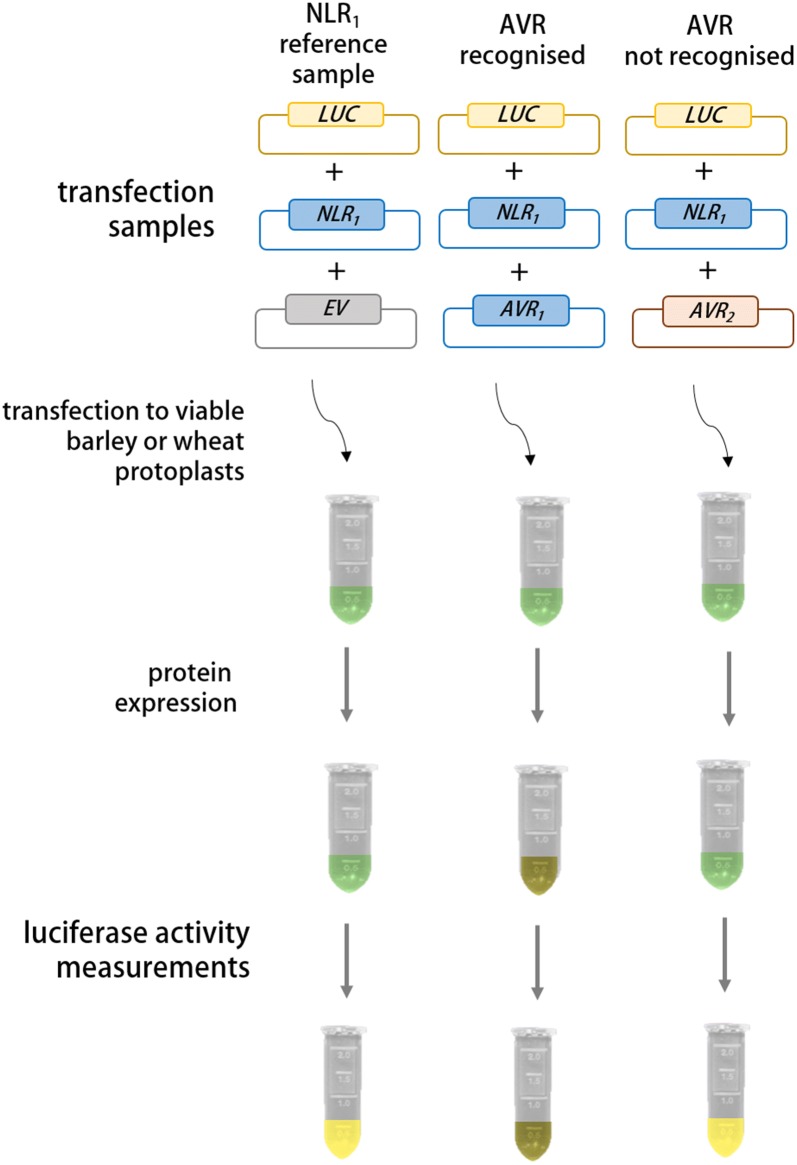
Schematic overview of experimental reasoning and expected outcomes. Viable wheat or barley protoplasts are transfected with plasmid mixtures and luciferase activity is determined as proxy for cell viability upon protein expression directed by transfected gene constructs. Reference sample contains constructs for *LUC*, *NLR* and *EV* to quantify reference luciferase activity when the *NLR* alone is overexpressed. In comparison to the reference samples, luciferase activity is expected to decrease only upon expression of matching NLR and AVR proteins (here NLR1 and AVR1)

To determine if mesophyll protoplasts of cereals can also be used for testing interspecies functionality of NLR/AVR pairs, we chose to focus on the Sr50/AvrSr50 and MLA1/AVR_A1_ pairs: The NLR encoded by *Sr50* from rye confers race-specific disease resistance to the wheat stem rust pathogen *Pgt* by the recognition of *Pgt AvrSr50* [20, 21]. Sr50 recognises AvrSr50 and the AvrSr50_RKQQC_ variant that differs from AvrSr50 by nine aa. One of these nine aa differences is located within the signal peptide (SP) region of AvrSr50. The virulent *Pgt* race QCMJC expresses AvrSr50_QCMJC_, which differs from AvrSr50 by 12 aa of which two are encoded in the signal peptide (SP) region [20]. The barley NLR MLA1 recognises AVR_A1_ for resistance to isolates of the powdery mildew fungus *Blumeria graminis* f. sp. *hordei* (*Bgh*) that carry *AVR*_*a1*_ [8, 17]. The AVR_A1_-V1 variant only differs by two aa from AVR_A1_ [8]. In barley protoplasts, co-expression of *Mla* and matching *AVR*_*a*_ can quantify MLA/AVR_A_-specific cell death [17]. Here, we determined if MLA1 could also act as a functional NLR in wheat. For this, we isolated wheat protoplasts and co-transfected the isolated cells with cDNAs of *AVR*_*a1*_ variants lacking SP and *Mla1*. Simultaneously, we tested if our method can be used to quantify death induced by the NLR-mediated recognition of AVRs from an unrelated pathogen. For this, we assessed death of wheat protoplasts transfected with cDNAs of *AvrSr50* effector variants lacking SP and *Sr50*. We utilised LUC activity as a proxy for cell viability [25]. Diminished LUC activity upon *AVR* transfection indicates *AVR*-specific cell death (Fig. 1). As such, we included a reference sample, which provides a read-out on LUC activity in the absence of an *AVR*. This reference sample consisted of *LUC* reporter, empty vector (*EV*) and *NLR* constructs of interest transfected into protoplasts from plants lacking the resistance specificity of interest (Table 1: sample 1 and sample 7). In the test samples, the *EV* construct was substituted by the plasmid encoding the *AVR* of interest (Table 1: sample 2, sample 10 and 11). For recognition specificity, we included a variant *AVR* construct not recognised by the specific *NLR* of interest. This effector variant is encoded by a virulent pathogen isolate (*AVR* control samples, Table 1: sample 3, *AVR*_*a1*_-*V1* substitutes for *AVR*_*a1*_; and sample 12, *AvrSr50*_*QCMJC*_ substitutes for *AvrSr50/AvrSr50*_*RKQQC*_) [8, 17, 20]. We tested AVR-mediated reduction of LUC in the presence or absence of the specific NLR of interest. For this, we substituted the *NLR* of interest by an alternative *NLR* (*NLR* control sample, Table 1: samples 4–6, *Mla1* substitutes for *Sr50* and samples 8 and 9, *Sr50* substitutes for *Mla1*). In total, we performed the experiment four times independently.

**Table 1:** Setup for measuring luciferase activity as proxy of cell death mediated by *AVR*_*a1*_ through recognition by *Mla1* and *AvrSr50* by recognition through *Sr50*

Sample	1	2	3	4	5	6	7	8	9	10	11	12
Reporter construct	LUC	LUC	LUC	LUC	LUC	LUC	LUC	LUC	LUC	LUC	LUC	LUC
NLR construct	*Mla1*	*Mla1*	*Mla1*	*Mla1*	*Mla1*	*Mla1*	*Sr50*	*Sr50*	*Sr50*	*Sr50*	*Sr50*	*Sr50*
EV or AVR construct	EV	*AVR*_*a1*_	*AVR*_*a1*_-*V1*	*AvrSr50*	*AvrSr50*_*RKQQC*_	*AvrSr50*_*QCMJC*_	*EV*	*AVR*_*a1*_	*AVR*_*a1*_-*V1*	*AvrSr50*	*AvrSr50*_*RKQQC*_	*AvrSr50*_*QCMJC*_

**Fig. 2:**
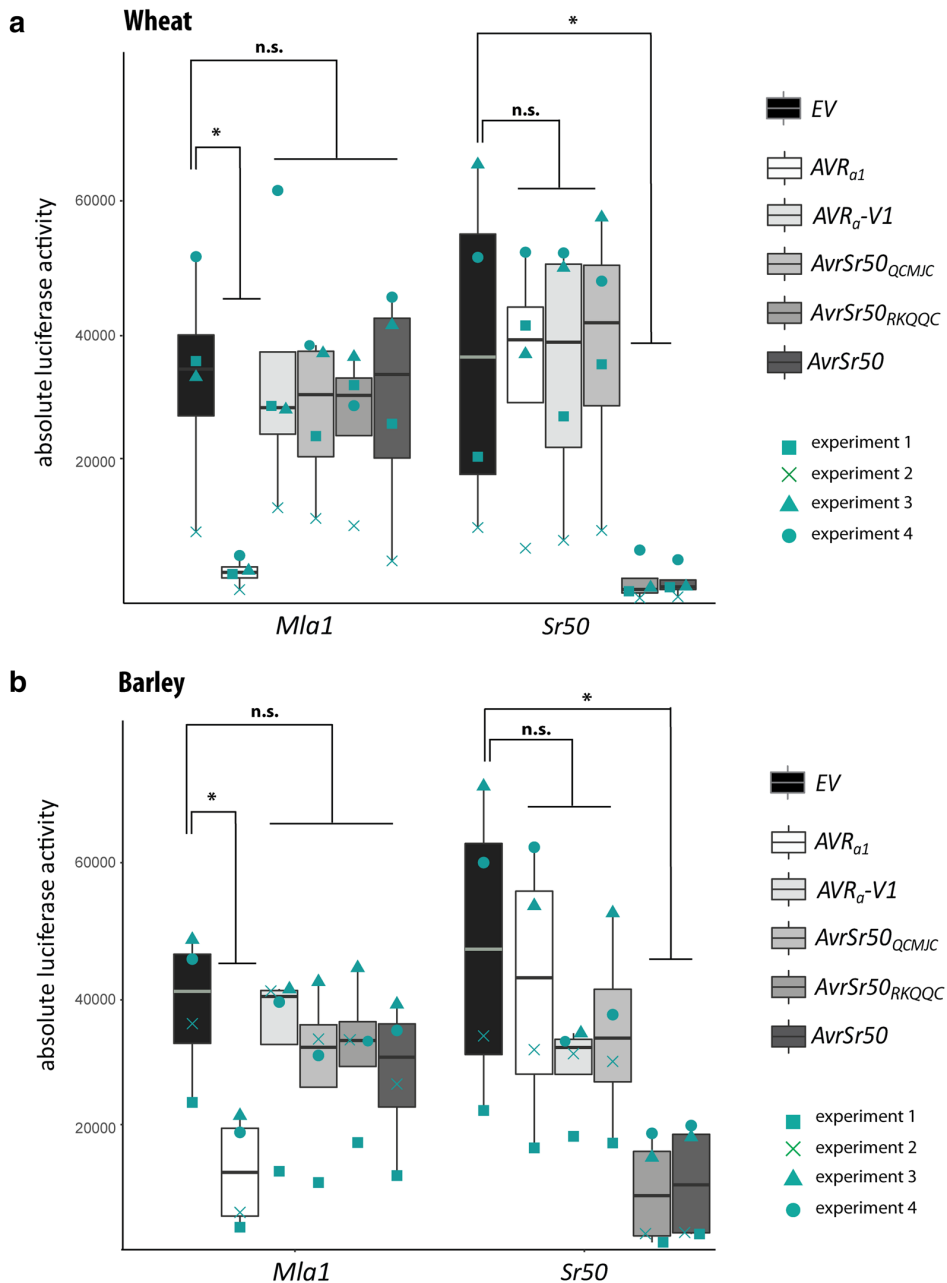
Results of example setup transfection (Tables 2 and 3) into wheat (**a**) and barley (**b**) mesophyll leaf protoplasts based on four biological replicates. Isolated protoplasts were transfected with *pUBQ:luciferase* and either a *pIPKb002* empty vector (*EV*) control or *pIPKb002* vector with cDNAs of *AVR*_*a1*_, *AVR*_*a1*_-*V1*, *AvrSr50*_*WT*_, *AvrSr50*_*RKQQC*_, *AvrSr50*_*QCMJC*_ all lacking respective signal peptides together with either *Mla1* or *Sr50*. Luciferase activity was determined 16 h post transfection as proxy for cell death. * indicate significant differences in luciferase measurements (**a**, **b**, non-parametric distribution). Calculated Kruskal–Wallis P values were as follows: **a**: P = 0.005261, **b**: P = 0.02896. n.s. not significant (P > 0.05). Experiment was performed four times independently with different plant material used each day and all values (Tables 2 and 3) obtained in the full biological replicates are indicated in turquoise: square; Experiment 1, cross: Experiment 2, triangle: Experiment 3, dot: Experiment 4

Similar to the results obtained in barley, exchange of *EV* to *AVR*_*a1*_ led to a significantly (P < 0.05, Kruskal–Wallis) reduced LUC activity in wheat cells when *Mla1* was co-expressed, but not when *Mla1* was exchanged to the in-wheat functioning NLR *Sr50* (Table 2, Fig. 2). LUC activity was not significantly different from the EV sample when *EV* was replaced by *AVR*_*a1*_-*V1*, a variant expressed by *Bgh* isolates virulent on *Mla1* barley lines. In turn, in comparison to the *EV* control, *AvrSr50* and its avirulent variant *AvrSr50*_*RKQQC*_ [20] significantly reduced LUC activity of wheat protoplasts when co-expressed with *Sr50* but not when co-expressed with *Mla1* (Table 2, Fig. 2a). LUC activity was statistically not significantly different when *EV* was replaced by *AvrSr50*_*QCMJC*_, a *AvrSr50* variant encoded by *Pgt* that escapes Sr50 recognition [20]. Similarly, in barley cells, co-expression of *AvrSr50* or *AvrSr50*_*RKQQC*_ [20] together with *Sr50* but not *Mla1* lead a significantly reduced LUC activity (Table 3, Fig. 2b).

**Table 2:** Luciferase (LUC) measurements obtained in independent experiments after transfection of wheat protoplasts

Experiment	*Mla1* reference sample 1	Sample 2	Sample 3	Sample 4	Sample 5	Sample 6	*Sr50* reference sample 7	Sample 8	Sample 9	Sample 10	Sample 11	Sample 12
	*Mla1*						*Sr50*					
	*EV*	*AVR*_*a1*_	*AVR*_*a1*_ *-V1*	*AvrSr50*	*AvrSr50*_*RKQQC*_	*AvrSr50*_*QCMJC*_	*EV*	*AVR*_*a1*_	*AVR*_a1_-V1	*AvrSr50*	*AvrSr50*_*RKQQC*_	*AvrSr50*_*QCMJC*_
1												
LUC measurement	35605	4001	29003	26349	32102	24521	21382	40966	27441	2042	1438	35195
Relative to reference	1	0.11	0.81	0.74	0.90	0.69	1	1.92	1.28	0.10	0.07	1.65
2												
LUC measurement	10208	1676	13855	5942	11176	12265	10849	7831	9012	613	439	10,481
Relative to reference	1	0.16	1.36	0.58	1.09	1.20	1	0.72	0.83	0.06	0.04	0.97
3												
LUC measurement	33215	4487	28457	41008	36279	36824	64846	36707	49537	2126	1974	57030
Relative to reference	1	0.14	0.86	1.23	1.09	1.11	1	0.57	0.76	0.03	0.03	0.88
4												
LUC measurement	51137	6750	61044	45179	29037	38032	51035	51858	51753	6128	7560	47560
Relative to reference	1	0.13	1.19	0.88	0.57	0.74	1	1.02	1.01	0.12	0.15	0.93

Results are shown for transfections 1–12 into wheat mesophyll leaf protoplasts

To compare between independent experiments, values relative to the respective reference samples (reference samples = 1) were calculated

**Table 3:** Luciferase (LUC) measurements obtained in independent experiments after transfection of barley protoplasts

Experiment	*Mla1* reference sample 1	Sample 2	Sample 3	Sample 4	Sample 5	Sample 6	*Sr50* reference sample 7	Sample 8	Sample 9	Sample 10	Sample 11	Sample 12
	*Mla1*						*Sr50*					
	EV	*AVR*_*a1*_	*AVR*_*a1*_-*V1*	*AvrSr50*	*AvrSr50*_*RKQQC*_	*AvrSr50*_*QCMJC*_	EV	*AVR*_*a1*_	*AVR*_*a1*_-*V1*	*AvrSr50*	*AvrSr50*_*RKQQC*_	*AvrSr50*_*QCMJC*_
1												
LUC measurement	23100	4038	12557	11891	16925	10833	21818	16108	17877	3009	1741	16850
Relative to reference	1	0.17	0.54	0.51	0.73	0.47	1	0.74	0.82	0.14	0.08	0.77
2												
LUC measurement	35094	6294	39993	25829	32577	32680	33162	31062	30451	3236	3050	29268
Relative to reference	1	0.18	1.14	0.74	0.93	0.93	1	0.94	0.92	0.10	0.09	0.88
3												
LUC measurement	47886	21009	40278	37968	43520	41387	71156	52962	33572	17732	14650	51862
Relative to reference	1	0.44	0.84	0.79	0.91	0.86	1	0.74	0.47	0.25	0.21	0.73
4												
LUC measurement	44916	18472	38312	33994	32367	30186	59554	61879	32341	19510	18326	36396
Relative to reference	1	0.41	0.85	0.76	0.72	0.67	1	1.04	0.54	0.33	0.31	0.61

Results are shown for transfections 1–12 into barley mesophyll leaf protoplasts

To compare between independent experiments, values relative to the respective reference samples (reference samples = 1) were calculated

**Fig. 3:**
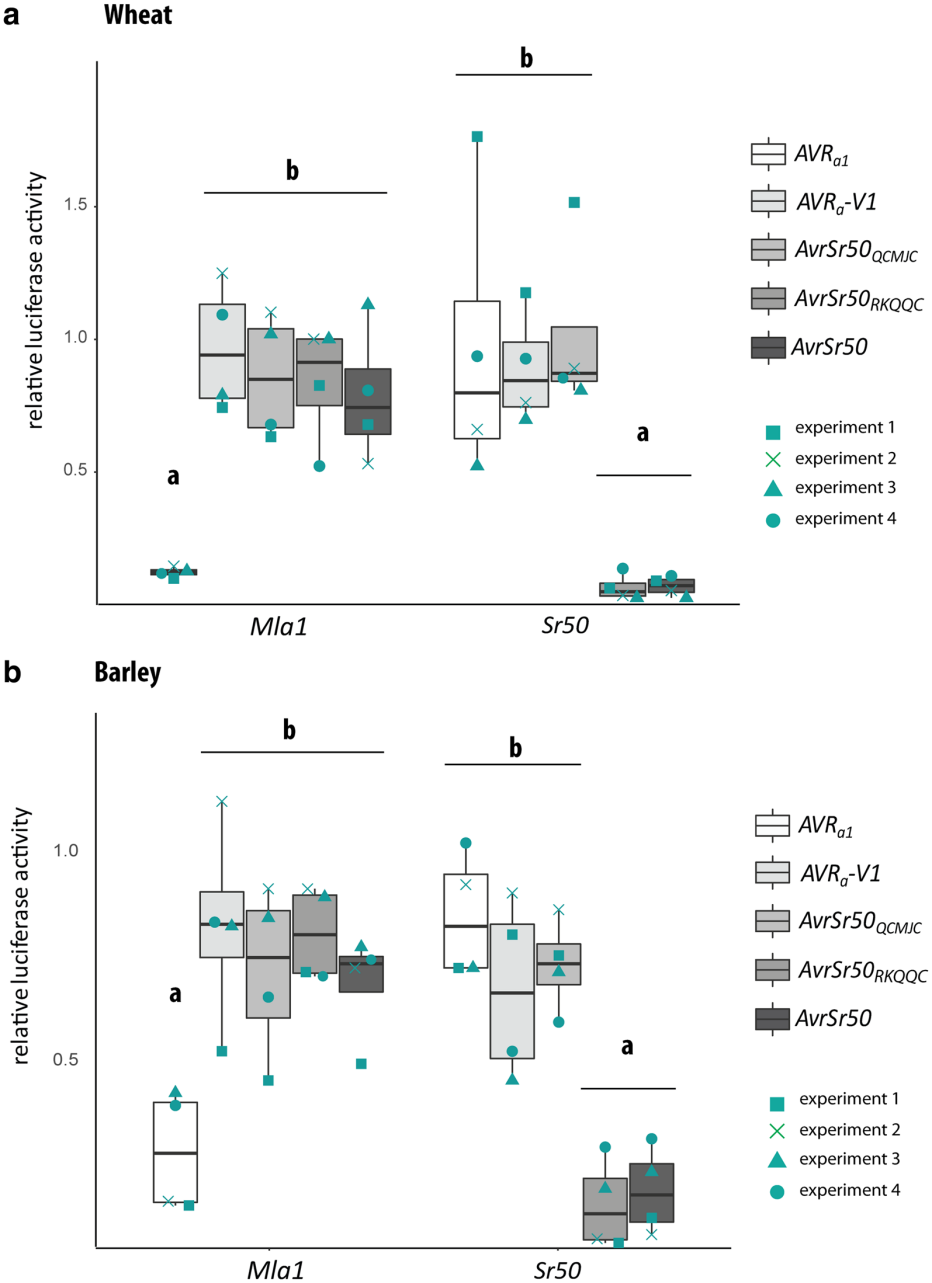
Results of example setup transfection (Tables 2 and 3) into wheat (**a**) and barley (**b**) mesophyll leaf protoplasts based on four biological replicates. Isolated protoplasts were transfected with *pUBQ:luciferase* and either a *pIPKb002* empty vector (*EV*) control or *pIPKb002* vector with cDNAs of *AVR*_*a1*_, *AVR*_*a1*_-*V1*, *AvrSr50*_*WT*_, *AvrSr50*_*RKQQC*_, *AvrSr50*_*QCMJC*_ all lacking respective signal peptides together with either *Mla1* or *Sr50*. Luciferase activity was determined 16 h post transfection as proxy for cell death. Differences amongst all transfection samples were assessed by analysis of variance and subsequent Tukey post hoc test of luciferase measurements normalised to the *EV* sample for each *NLR* construct (EV = 1). Observed P values were as follows: **a** P = 1.594e−06, **b** P = 1.573e−07. Samples marked by different letters differ significantly (P < 0.05) in the Tukey test. Experiment was performed four times independently with different plant material used each day and all values (Tables 2 and 3) obtained in the full biological replicates are indicated in turquoise; square: Experiment 1, cross: Experiment 2, triangle: Experiment 3, dot: Experiment 4

In total, the experiments were performed four times on different days with protoplasts obtained from plants grown independently for each biological replicate (Tables 2, 3, Fig. 2) and we observed that absolute LUC measurements of the same transfection sample varied up to sixfold between individual experiments (Tables 2, 3, Fig. 2). This variability in LUC measurements between biological replicates might depend on the quality of the transfected protoplasts, the integrity of the plasmid preparations, the routine of the researcher performing the individual experiments, or other parameters. To account for variation of absolute LUC values between independent experiments and for the putative autoactivity of overexpressed *NLR*, we analysed relative LUC values normalised to the respective NLR reference sample in the particular experiment [8, 17] (Tables 2, 3, Fig. 3). Reduced relative LUC activity of matching AVR/NLR transfection samples differ significantly from all control samples in the Tukey post hoc test (p < 0.05, Fig. 3).

## Discussion

Here we present a method that can be deployed to screen candidate NLR/AVR pairs and to verify matching NLR/AVR pairs directly in the barley and wheat hosts (Fig. 2, Tables 2, 3). The *NLR* interspecies transfer with ensuing cell death activity mediated by barley MLA1 in wheat and, conversely, the cell death mediated by the wheat stem rust NLR Sr50 in barley (Fig. 2) demonstrates the approach to be suitable for functional assays of NLR-mediated cell death execution in other cereal species.

Cell death measurement upon protoplast transfection with an *AVR* gene may also be employed to screen *AVR* candidates in resistant lines even when the host *NLR* has not been molecularly isolated. We have described this previously in barley [8] but have not performed corresponding experiments in wheat. In barley we found a higher variation in cell death measurements in a setup where the *NLR* is not overexpressed. Consequently, some *AVR* effectors might escape detection by the latter test system. One reason could be a requirement of certain pathogen-induced *NLR* levels for AVR recognition and subsequent cell death initiation. For example a clear change in *Mla* transcript was detected upon pathogen inoculation [26]. However, we have not tested whether pathogen inoculation of resistant plants prior to protoplast preparation renders protoplasts more sensitive to cell death following *AVR* transfection.

Like most protocols for identifying *AVR/NLR* pairs, the method described here also relies on the common acceptance that NLR-mediated disease resistance depends on AVR-specific NLR-mediated host cell death. Still, examples of race-specific disease resistance have been described in dicotyledonous and monocotyledonous plant species in which NLR function does not rely on triggering cell death to mediate pathogen resistance [27–30]. As such, the ability of these NLRs to recognise pathogen effectors might not be detected using the current protocol or any alternative methods for assessing AVR/NLR-mediated cell death. However, in the immune response without cell death conferred by the potato NLR Rx to potato virus X, the receptor has the potential to initiate cell death when the avirulence effector was constitutively over-expressed, i.e. was uncoupled from viral replication [27].

The transfection of protoplasts derived from the natural host of the pathogen represents a rapid alternative to the generation of stable transgenic cereal plants for testing AVR/NLR pairs. Our protocol has been optimised to ensure the efficient transfection of binary plasmids. Thus, conventional binary expression vectors compatible for potential generation of stable transgenic lines by *Agrobacterium*-mediated plant transformation can be used. One suitable Gateway-compatible vector is the pIPKb002 vector, deployed here [31]. pIPKb002 encodes the bacterial spectinomycin selection marker gene suitable for the transformation of cereals using the hyper-virulent *A. tumefaciens* strain AGL1. Smaller-sized plasmids might be used (see Quality, size and nature of plasmid DNA below) so long as an appropriate promotor sequence for the expression of each construct in cereal mesophyll cells is employed. For monocots such as barley and wheat, the *ZmUBQ* promoter ensures optimal expression of genes of interest [32]. Plant material for the protocol described here can be obtained within 1 week after sowing seeds. Isolation and transfection of protoplasts can be performed within a single working day and the results of AVR-specific NLR-mediated cell death are obtained the morning after protoplast transfection.

When transfecting a single *pZmUBQ:GFP* reporter plasmid, we observe GFP expression in 25% to 50% of transfected barley protoplasts and 30% to 70% of transfected wheat protoplasts as determined by fluorescence microscopy. This might account for the higher variance of relative LUC measurements in protoplasts obtained from barley cv. GP leaves when compared to LUC activities obtained from transfected wheat cv. Svevo leaves (Table 2 and Table 3, Fig. 2). Still, only four biologically independent transfections were sufficient to identify/validate the matching AVR/NLR pairs in both barley and wheat.

## Conclusions

Here we provide quantitative cell death measurements mediated by the recognition of transiently expressed *AvrSr50* from the basidiomycete *Pgt* through the matching NLR *Sr50* and by the detection of transiently expressed *AVR*_*a1*_ from the ascomycete *Bgh* through barley *Mla1,* both in wheat and in barley mesophyll protoplasts. Using the method described here, we depict how mesophyll protoplasts derived from barley and wheat leaves, and possibly leaves from other cereals, can be transfected and screened for the identification and verification of pathogen effector candidates derived from two unrelated host-adapted fungal pathogens. Our results suggest that this method can be applied for the assessment of NLR function in diverse host cultivars or other cereal plant species.

## Methods

### Plant growth and tissue selection

**Fig. 4:**
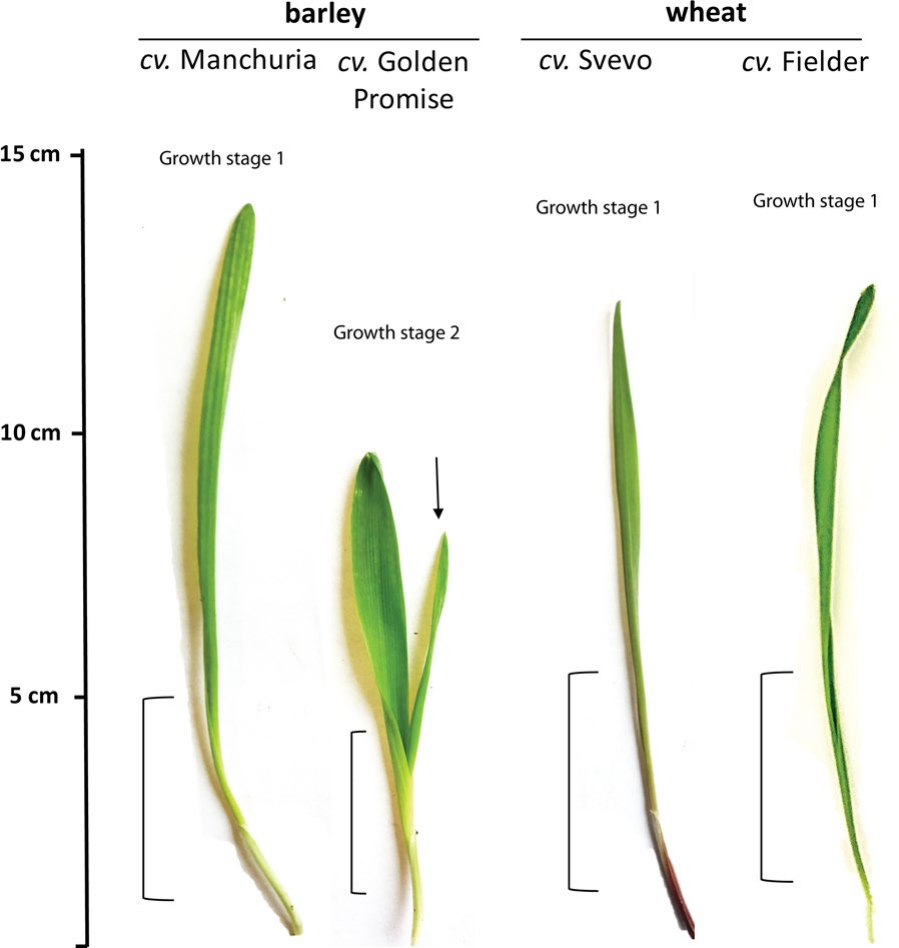
Selection of leaf tissue for protoplast isolation. Barley and wheat plants are grown to a size of 9–15 cm from base to tip. Growth stage 1 represents cultivars (*cv.*) that reach this seedling size by growing a long first leaf (e.g. barley *cv.* Manchuria, wheat *cv.* Svevo and *cv.* Fielder). Growth stage 2 represents cultivars that reach this seedling size by growing a short first leaf and a second leaf (e.g. barley *cv.* Golden Promise). Arrow indicates the leaf optimal for mesophyll protoplast isolation of second leaf stage cultivars. Brackets indicate the respective leaf region to be selected for isolation of mesophyll protoplasts

The size of the seedling is critical for the isolation of protoplasts that are optimal for transfection. At 19 °C, 70% relative humidity, and with a 16 h photoperiod, wheat and barley seedlings grow to a total size of 9–15 cm from base to tip (Fig. 4) within 7 to 9 days. Care was taken to keep soil moist at all times. Under these growth conditions, two seedling growth stages can be observed (Fig. 4). For example, barley *cv.* Manchuria, wheat *cv.* Svevo and wheat *cv.* Fielder reach the optimal size at growth stage 1, which is characterised by the growth of the first true leaf without the emergence of a second leaf. In turn, barley *cv.* Golden Promise (GP) reaches the optimal size at growth stage 2, characterised by the growth of a short primary leaf and the emergence of a second leaf. Here, the second true leaf was selected. The tissue of the youngest leaf just above the seedling coleoptile was chosen for protoplast isolation (Fig. 4). Our attempts to transfect protoplasts of older tissue or protoplasts obtained from primary GP leaves remained unsuccessful as determined by the deficiency or high variation of luciferase activity after transfection with a luciferase reporter gene.

### Quality, size and nature of plasmid DNA

In this study, we chose the Gateway-compatible vector pIPKb002 [31] for the expression of *NLR* and *AVR* genes. The vector can also be used for the generation of stable transgenic lines by *Agrobacterium*-mediated plant transformation and its use here avoids the need to generate smaller-sized plasmids suitable only for protoplast transfection experiments. We have deposited the *pZmUBQ:LUC* reporter plasmid used here [33] (Addgene ID: 132360), provide a small size p*ZmUBQ* encoding Gateway-compatible empty vector plasmid [34] (Addgene ID: 132358) and the corresponding vector variants encoding *Mla1* (Addgene ID: 132355), *AVR*_*a1*_ (lacking SP, Addgene ID: 132356) and *AVR*_*a1*_-*V1* (lacking SP, Addgene ID: 132357) cDNAs. All constructs have been successfully used in the here described assay.

We here aimed for the co-transfection of three conventional binary expression vectors into wheat and barley protoplasts. For this, we found that pure and highly concentrated plasmid DNA is required. Our attempts to transfect protoplasts with plasmids obtained by conventional *Escherichia coli* plasmid miniprep kits remained unsuccessful as determined by the deficiency of luciferase activity after transfection with a luciferase reporter plasmid obtained by the NucleoSpin Plasmid Miniprep kit (Macherey-Nagel). However, we obtained highly pure and concentrated plasmid DNA from 350 ml of overnight cultures of *E. coli* harbouring the constructs of interest using the NucleoBond^®^ Xtra Maxi Plus (Macherey-Nagel, catalogue number 740416.10) or HiSpeed Plasmid Maxi (Qiagen, catalogue number 12662) plasmid extraction kits. To limit consumable costs, we frequently use the kit manufactured by Macherey-Nagel but are unable to detect major qualitative differences between kits from different manufacturers.

### Preparation of plasmids

For each construct, five ml of sterile LB broth including appropriate antibiotic in a sterile 50 ml plastic tube were inoculated with the *E. coli* strain carrying the construct of interest. The starter culture was incubated overnight at 37 °C with shaking at 250 rpm. For each construct, 350 ml of sterile LB broth including appropriate antibiotic in a sterile 2 l conical flask were inoculated with the 5-ml starter culture. The main cultures were incubated overnight at 37 °C with shaking at 250 rpm. HiSpeed Plasmid Maxi (Qiagen, catalogue number 12662) or NucleoBond^®^ Xtra Maxi Plus (Macherey-Nagel, catalogue number 740416.10) plasmid extraction kits are suitable for the isolation of highly concentrated plasmids. The kits were used according to the manufacturers’ instructions with the following modification: For the last step, instead of elution buffer, 300 µl of nuclease free water were used for elution of plasmids from the membrane. Water is used instead of elution buffer, as the latter contains pH 7–7.5 buffer components, which can interfere with the pH 5.7 buffer components, used for plasmid transfection (Table 4). The concentration and quality of isolated plasmids were assessed using a Nanodrop spectrophotometer. Barrier tips and nuclease-free water was used to dilute all constructs to 1 µg/µl. We have observed low concentrations (< 1 µg/µl) for some *NLR* gene containing expression vectors after large scale plasmid preparations. In such cases, all plasmid preparations were diluted to 500 ng/µl. Plasmids were frozen at − 20 °C as 100-µl aliquots until immediate use.

**Table 4:** Quantities of stock solutions and reagents required for the preparation of working buffers for wheat and barley mesophyll leaf protoplast isolation and transfection

Reagents/stock solutions	Protoplast isolation buffer^a^	Wash buffer^a^	Transfection buffer 1^a^	Transfection buffer 2^a^	Regeneration buffer^a^
0.8 M Mannitol	7.5 ml		12.5 ml	1.25 ml	11.25 ml
PEG 4000				2 g	
0.1 M MES pH 5.7	1 ml	1 ml	1 ml		600 µl
2 M KCl	100 µl	125 μl			150 µl
5 M NaCl		1.54 ml			
1 M MgCl_2_			375 µl		
Cellulase R10	150 mg				
Macerozyme R10	50 mg				
Heat to 55 °C for 10 min, cool to room temperature					
1 M CaCl_2_	100 µl	6.25 ml		500 µl	
BSA	10 mg				
ddH_2_O	Up to 10 ml	Up to 50 ml	Up to 25 ml	Up to 5 ml	Up to 15 ml
	Filter sterilize	Filter sterilize			Filter sterilize

^a^Volumes suitable for 12 independent transfections

### Buffers

One hundred milliliters of 1 M CaCl_2,_ 5 M NaCl, 2 M KCl 1 M MgCl_2_ and 0.1 M MES pH 5.7 stock solutions were prepared with double-deionised water, filter sterilised and stored at 4 °C. On the day of transfection, 50 ml 0.8 M mannitol and working stock solutions (Table 4) were freshly prepared using dilutions of stock and mannitol solutions in double deionised water. Barrier tips were used for the preparation of all solutions. The volume for mannitol and each working solution required depends on the number of transfections anticipated. The amounts of reagents indicated (Table 4) are suitable for 12 individual transfections and can be adjusted accordingly.

### Preparation of leaf tissue (Fig. 5)

One barley or wheat seedling per transfection was used for epidermal peeling. Each leaf was cut with a razor blade and placed on a soft surface with the adaxial side facing up. Razor blade was placed at the vertical center of the leaf, and moderate pressure applied to cut through upper epidermis and mesophyll cells (step 1, Additional file 1: Video). Caution was taken to not cut through lower epidermis. The tip of the leaf was gently bent down to detach abaxial epidermis and peel epidermis from the base half of the leaf (step 2, Additional file 1: Video). The removal of abaxial epidermis is facilitated by the selection of young leaf tissue and on plants grown in moist soil (see ‘Plant growth and tissue selection’ above). Using a razor blade, the vertical centre of base half of the leaf was cut through with detached abaxial epidermis and leaf base was placed into protoplast isolation buffer (step 3). When all leaves were peeled and cut, the tube containing leaves in protoplast isolation buffer was placed into a rack and tube was opened. The rack with the open tube was placed into a desiccator and continuous vacuum was applied for 45 min to allow the buffer to penetrate the intracellular space. The vacuum pump (Vacuubrand MZ 20 at 2.4 m^3^/h) remained turned on during the whole incubation time (step 4). Vacuum was released over a period of 15 s and the tube containing leaf tissue was closed (step 5).

**Fig. 5:**
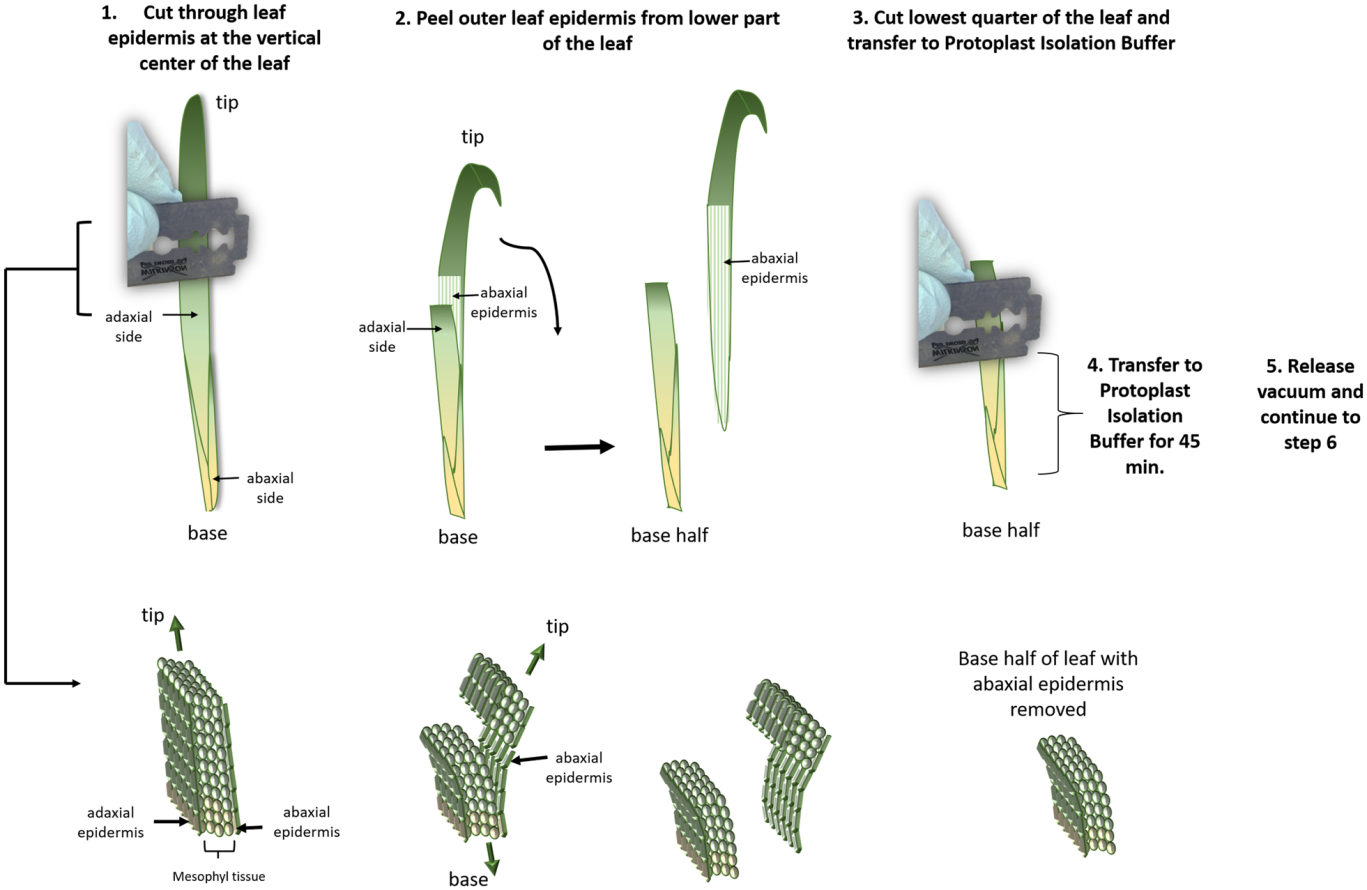
Schematic representation of tissue preparation for isolation of mesophyll protoplasts from wheat or barley leaf. Step 1: Selected leaf is placed onto soft surface adaxial leaf facing upwards. Using a razor blade, gentle pressure is applied at the middle part of the leaf to cut through adaxial epidermis and mesophyll tissue without cutting through abaxial epidermis. Step 2: Tip of the leaf is pulled down to remove abaxial epidermis from the base half of the leaf. Step 3: The base half of the leaf with abaxial epidermis removed, is cut in half and bottom part is transferred into Protoplast Isolation Buffer in step 4 and buffer is vacuum infiltrated in step 5

### Protoplast isolation (Fig. 6)

The tube with protoplast isolation buffer containing leaf tissue was wrapped in aluminium foil and placed horizontally onto shaker and incubated for 3 h at room temperature in the dark with shaking at 60 rpm (step 6). After the 3 h incubation period, one volume (10 ml) of wash buffer was added to protoplast isolation buffer containing leaf tissue (step 7). A 100-µm nylon cell strainer was submerged in ~ 5 ml of wash buffer before placing cell strainer into a fresh open 50 ml tube. Protoplast isolation buffer containing leaf tissue was slowly decanted into cell strainer, there both Falcon tubes were held at 45° angles. Flow-through contained isolated protoplasts (step 8). Filtered buffer containing leaf protoplasts was slowly decanted into two 30-ml round-bottom centrifuge tubes and tubes were centrifuged for 3 min at 100*×g* to collect protoplasts at the bottom of the tubes (step 9). Using a 5-ml pipette, supernatant was removed. So as not to disturb the protoplast pellet, approximately 500 µl of supernatant were left in each tube and used to resuspend protoplasts by swirling the round bottom tubes (step 10). Using a 5 ml pipette, 5 ml wash buffer were added into each round-bottom tube by holding the round-bottom tube at a 45° angle and pipetting buffer down the wall of the tube. Caution was taken to not pipette buffer directly onto isolated protoplasts (step 11).

**Fig. 6:**
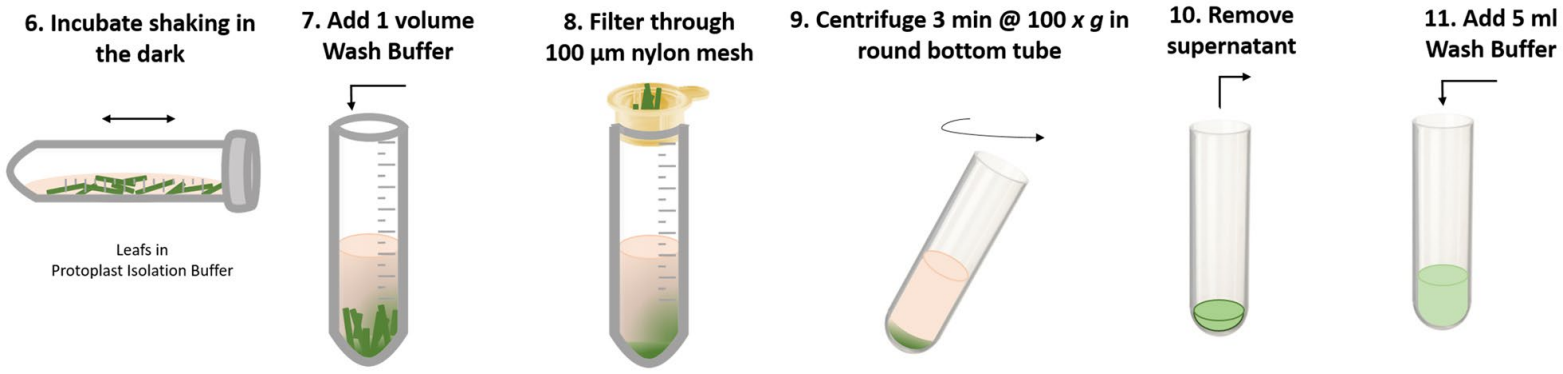
Visual overview of steps for the protoplast isolation from wheat or barley leaves. Step 6: After vacuum infiltration, tube containing leaf tissue is incubated for 3 h at room temperature with 60 rpm shaking in the dark. Step 7: One volume of Wash Buffer is added to 1 volume of Protoplast Isolation Buffer containing leaf tissue. Step 8: Diluted buffer containing leaf tissue is filtered through a pre-moistened 100 µm—nylon cell strainer into a fresh tube. Step 9: Flow through containing protoplasts is centrifuged in round bottom tube at 100 *x g* for 3 min. Step 10: Supernatant is removed using a pipette. Step 11: Wash buffer is added to protoplast pellet

### Adjustment of protoplast density and preparation of plasmid for transfection (Fig. 7

0.5 ml of wash buffer containing protoplasts were removed and kept for the determination of cell concentration (step 12). Round-bottom tube containing remaining isolated protoplasts in wash buffer were left to settle protoplasts for up to 45 min in the dark. Caution was taken to not leave protoplasts in wash buffer for more than 45 min, as level of recovered viable protoplasts decreased with extended incubation times in wash buffer at this step (step 13). A concentration of approximately 3.5 × 10^5^ protoplasts/ml in the absence of any cell debris contaminants was found to be suitable for the following transfection assay. Cell numbers can be determined using a standard hemocytometer (for example BRAND counting chamber, SIGMA cat. no. BR717810) and cell concentrations are calculated according to the instructions of the hemocytometer’s manufacturer. A microscopic inspection can also be employed to determine whether the protoplast solution is free of cell debris. The here described release of mesophyll protoplasts after epidermal peeling should result in a protoplast solution without or minimal cell debris (Fig. 8a). If this is obtained consistently, experienced users might also consider using optical density readouts to determine protoplast concentration at this step. An OD_600_ = 0.4 corresponds to approximately 3.5 × 10^5^ protoplasts/ml (Fig. 8b). Here we determined the OD_600_ of wash buffer containing protoplasts by mixing 0.5 ml wash buffer containing protoplasts (see step 12) with 0.5 ml wash buffer in a 1 ml cuvette and 1 ml wash buffer was used as blank. Protoplasts used for OD_600_ measurement were discarded (step 14).

**Fig. 7:**
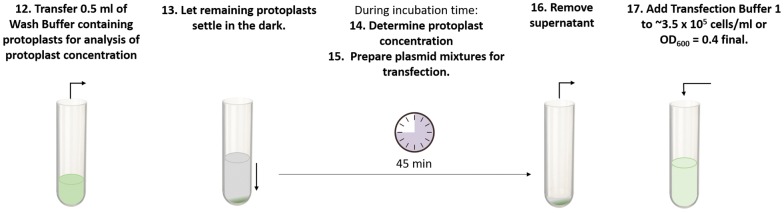
Overview of steps for the adjustment of protoplast density and preparation of plasmid for transfection. Step 12: 0.5 ml of Wash Buffer containing protoplasts is transferred to cuvette. Step 13: Round bottom tube containing isolated protoplasts in Wash Buffer is placed in dark environment to let protoplasts settle for 45 min. Step 14: Protoplast concentration is determined. Step 15: Preparation of plasmid transfection mixtures. Step 16: Wash Buffer is removed from protoplast pellet using a pipette. Step 17: Transfection Buffer 1 is added to protoplast pellet for a calculated final OD_600_ = 0.4

**Fig. 8:**
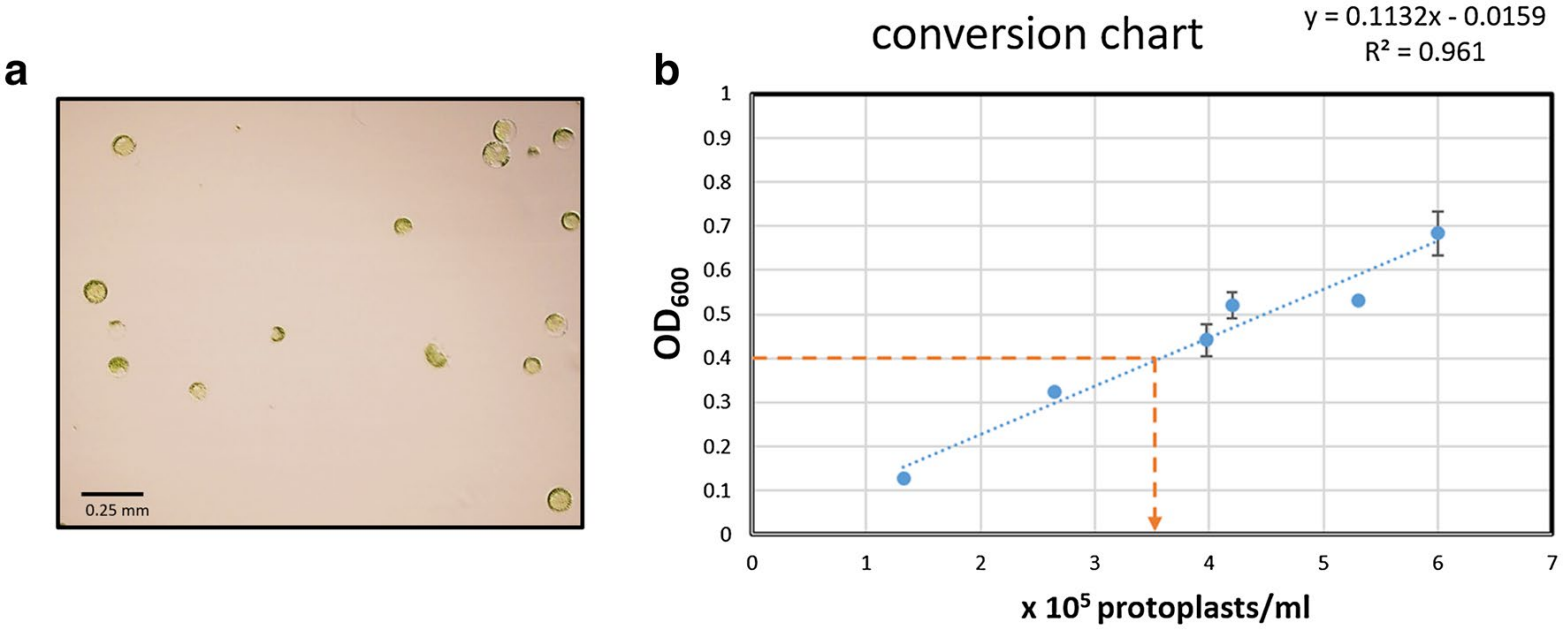
Correlation between OD_600_ and protoplast concentration in protoplast suspensions without cell debris. Isolation of mesophyll leaf protoplasts from barley and wheat should result in suspensions of protoplast free of detectable cell debris (**a**, protoplast of wheat cv. Fielder). In protoplast suspensions a correlation between the concentration of protoplasts and an optical density measurement allows quantification of protoplast concentration by OD_600_ measurements in step 15 of this protocol. An OD_600_ = 0.4 corresponds with 3.5 × 10^5^ protoplasts/ml (**b** yellow lines), which is the preferred protoplast concentration for subsequent transfection steps

For the preparation of plasmid mixtures aliquots of the luciferase reporter construct and *EV*, *AVR* and *NLR* constructs were thawed. Eight µl of the LUC reporter construct [33], 12 µl of the NLR constructs and 10 µl of the EV or AVR constructs (LUC/NLR/AVR ratio = 4:6:5) were mixed for each transfection (step 15). We found that the ratio of constructs within transfection samples depends on the molecular weight of each construct.

Using a 5-ml pipette, most of the supernatant in the round-bottom tube containing protoplasts was removed from the loose protoplast pellet. Round-bottom tube containing protoplast pellet was swirled to resuspend protoplasts in the remaining (~ 500 µl) wash buffer (step 16). Based on OD_600_ of wash buffer containing protoplasts, the volume needed to obtain a final OD_600_ = 0.4 was calculated. Using a 5-ml pipette, transfection buffer 1 was slowly added to a calculated final OD_600_ = 0.4. For this, the round-bottom tube was held at a 45° angle and buffer was pipetted against the wall of the tube but not directly onto the protoplasts (step 17).

### Protoplast transfection (Fig. 9)

Two ml low-bind tubes were labelled with transfection sample number (Table 1) and 300 µl of transfection buffer 1 containing protoplasts were transferred into each tube (step 18) using standard 1 ml barrier pipette tips. The entire plasmid mixture (30 µl) was pipetted directly into transfection buffer 1 containing protoplasts in sample tube 1 (step 19). Using standard 1 ml barrier pipette tips, 350 µl transfection buffer 2 was added immediately to the protoplast/plasmid mixture and the tube was closed. The solutions were mixed completely by inverting the tube at a rate of approximately 1 inversion/second (step 20). After 12 inversions, the buffers had mixed entirely, forming a homogeneous solution (step 21). Tube was placed into a rack and incubated for 15 min without disturbing the protoplasts (step 22). During these 15 min, steps 19 to 22 were repeated for the next 5 transfection samples consecutively (step 23). Starting with the first transfected tube, 2× 660 µl of wash buffer were pipetted into transfection tube 1 using a 1 ml pipette with standard tips and the lid of the tube was closed (step 24). By carefully inverting the tube eight times, the solutions were mixed completely, forming a homogeneous suspension (step 25). All six transfections were centrifuged together at 100×*g* for 3 min and a 1 ml pipette was set to 965 µl (step 26). After centrifugation, all centrifuged tubes were placed back into a rack. A pellet is not visible and protoplasts remained smeared along the side of the tube facing the outside of the centrifuge. Using standard 1 ml pipette tips, 1930 µl of the supernatant was removed by pipetting off 2× 965 µl from the side of the tube that faced the inside of the centrifuge. This step was repeated for the other five transfection samples (step 27). 965 µl of regeneration buffer was pipetted into each transfection tube and tubes were closed (step 28). All transfection sample tubes were placed into a rack and the rack was carefully wrapped in aluminum foil to avoid light stress during regeneration time. Protoplasts were regenerated by placing the wrapped rack into a 20 °C incubator at a 45° angle, keeping the rack stationary for 14 to 16 h (step 29). The next six samples were then transfected by starting from step 19.

**Fig. 9:**
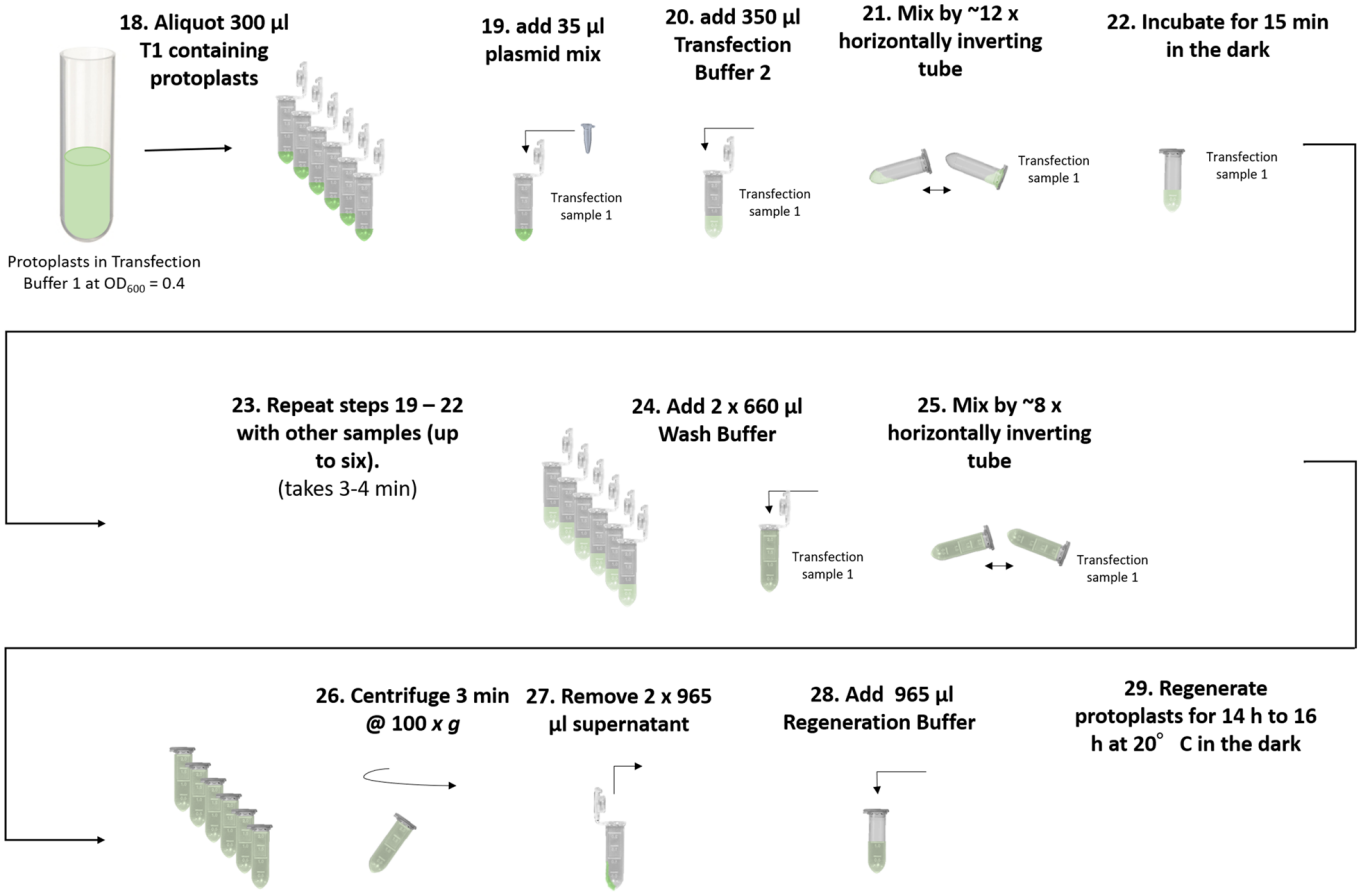
Overview of steps for the transfection of isolated protoplasts. Step 18: 300 µl Transfection Buffer 1 containing protoplasts are transferred to each transfection tube. Step 19: Using a pipette, plasmid transfection sample 1 is added to transfection sample tube 1 directly into Transfection Buffer 1 containing protoplasts. Step 20: 350 µl Transfection Buffer 2 is added to transfection sample tube. Step 21: Transfection sample tube is inverted 12 times. Step 22: Transfection sample tube is placed into rack in the dark. Step 23: Steps 19 to 22 are repeated with all other transfection samples one after another. Step 24: After 15 min incubation in the dark, 2× 660 µl of Wash Buffer is added to transfection sample tube. Step 25: Transfection sample tube is inverted 8 times. Step 26: All transfection sample tubes (up to six at the time) are centrifuged at 100×*g* for 3 min. Step 27: Using a pipette, 2× 965 µl are removed from all transfection sample tubes. Step 28: 965 µl of Regeneration Buffer is transferred into each transfection sample tube. Step 29: All transfection samples tubes containing protoplasts are regenerated at 20 °C in the dark for 16 h

### Protoplast recovery and determination of viable protoplasts by luciferase measurement (Fig. 10)

The first six transfections were centrifuged together at 1000*×g* for 3 min whilst setting a 1-ml pipette to 965 µl (step 30). All tubes were placed back in a rack. Protoplasts had formed a small pellet at the side of the tube facing the outside of the centrifuge. Using standard 1 ml pipette tips, 965 µl of the supernatant were pipetted off from the side of the tube that was facing the inside of the centrifuge. This step was repeated for the other five transfection samples (step 31). The protoplasts were then lysed for subsequent LUC activity measurements of cell extracts. For this, 200 µl of 2× cell culture lysis buffer (Promega E1531) was added into each of the first six transfection tubes and tubes were closed (step 32). Each of the first six transfection tubes was vortexed and placed on ice (step 33). The next six samples were then processed by starting from step 31. LUC activity of non-lysed protoplasts might be measured but we suggest the use of a buffer with a pH of 7.5 for optimal LUC enzyme activity. For LUC measurements, 50 µl of each transfection sample were transferred to wells of a standard white 96-well plate (Sigma-Aldrich, cat. no. CLS3922). LUC activity is measured by the addition of LUC substrate and resulting instantaneous light emission is to be measured directly after the addition of LUC substrate to the samples. Thus, a multichannel pipette was used for the addition of 50 µl LUC substrate solution (Promega E151A and E152A) into each well. Immediately thereafter, the 96-well plate was placed into a luminometer (the Berthold Centro LB 960 luminometer was used here) and LUC activity of each sample was measured for 1 s/well.

**Fig. 10:**
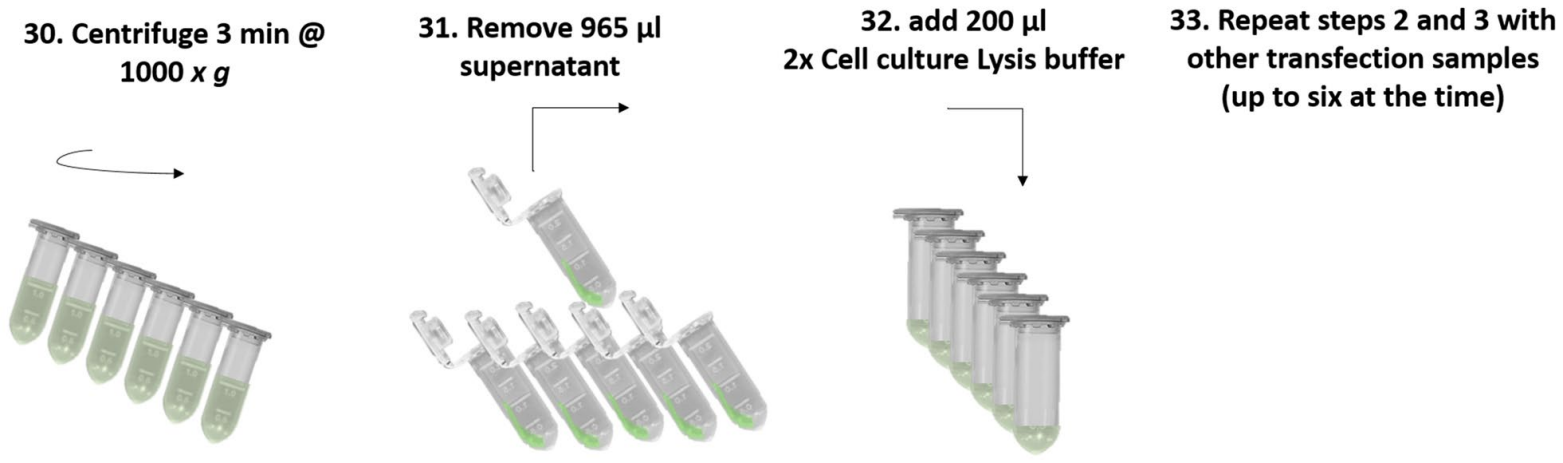
Overview of steps for protoplast recovery. Step 30: Up to six transfection samples tubes are centrifuged together at 1000 *x g* for 3 min. Step 31: Using a pipette, 965 µl of supernatant are remove from all transfection sample tubes. Step 32: 100 µl of 2× Cell Culture Lysis Buffer is transferred into each transfection sample tube. Step 33: Steps 30 to 32 are repeated for other transfection sample tubes

### Replication

For obtaining independent and reproducible data, we suggest to perform at least three fully independent replicates of the experiment (step 1 to step 33) on different days and using material of plants grown independently.

## Supplementary information

**Additional file 1.** Removal of abaxial leaf epidermis

## Data Availability

Raw data obtained for all independent experimental replicates are displayed in Tables 2, 3.

## Abbreviations

NLR: nucleotide-binding and leucine-rich repeats
AVR: avirulence
*Mla*: mildew locus A
*Bgh*: *Blumeria graminis* formae speciales *hordei*
*Pgt*: *Puccinia graminis* formae speciales *tritici*
LB: Luria-Bertani
LUC: firefly luciferase
ANOVA: analysis of variance
